# Evolution of Nrf2 Gene Expression in HIT-T15 β-Cells During Chronic Oxidative Stress and Glucose Toxicity

**DOI:** 10.1210/jendso/bvac178

**Published:** 2022-12-22

**Authors:** Tsehay Abebe, Lindsey Bogachus, Adithya Krishna Vegaraju, R Paul Robertson

**Affiliations:** Department of Internal Medicine, University of Washington, Seattle, USA; Department of Internal Medicine, University of Washington, Seattle, USA; Department of Internal Medicine, University of Washington, Seattle, USA; Department of Internal Medicine, University of Washington, Seattle, USA

**Keywords:** Nrf2 gene expression, beta cells, oxidative stress

## Abstract

**Context:**

Chronic exposure of pancreatic islets to elevated glucose levels causes progressive declines in beta cell *Pdx-1* and *insulin* gene expression, and glucose-induced insulin secretion. This has been shown to be associated with excessive islet reactive oxygen species and consequent damage to beta cell function, a process termed glucose toxicity. In short-term rodent in vivo studies, Nrf2 (Kelch-like ECH-associated protein 1:nuclear factor erythroid-derived-2 related factor complex) has been shown to play a central role in defending beta cells from oxidative damage via activation of antioxidant gene expression.

**Objective:**

The current studies were primarily designed to examine the behavior of Nrf2 gene expression during longer term exposure of beta cells to glucose toxicity.

**Methods and Results:**

We provide evidence that gene expression of Nrf2 in HIT-T15 cells, an insulin-secreting beta-cell line, undergoes a biphasic response characterized by an initial decrease followed by increased expression during prolonged culturing of these cells in a physiologic (0.8 mM) but not a supraphysiologic (16.0 mM) glucose concentration. This was associated with a slight rise in *HO-1* gene expression. *Pdx-1* and *insulin* mRNA levels also decreased but then stabilized in late passages of cells that had been cultured in low glucose concentrations.

**Conclusion:**

These complex events support the concept that Nrf2 gene expression plays an important regulatory role in defending beta cells during prolonged exposure to oxidative stress.

The beta cell line HIT-T15 was not used for studies of beta-cell function until the 1980s. Earlier passages of this cell line had not been saved so that eventually only passages in the low 60s were left. When our laboratory began using this line in the late 1980s, we took care to split and save back passages from thereon. For the current study we used the lowest passage available (P-70). Our previous publications have reported that the ability of this line to secrete insulin in a glucose-dependent manner underwent gradual deterioration if the cells underwent continuous culturing in media containing 16.0 mM glucose up to passage 90 [1]. Notably, the glucose dose–response curve for these cells is left shifted so that the median effective concentration for glucose is 2.0 mM and maximal at approximately 8.0 mM, which is only midmaximal for isolated islets. We interpreted the gradual loss in insulin response to be due to glucose toxicity because we could slow this deterioration if we cultured the cells in media containing a midmaximal concentration of 0.8 mM rather than using the supraphysiologic 11.1 mM concentration that had been used previously by others [2-13]. Additionally, we have shown that these functional changes in late passages of HIT-T15 cells are not due to dedifferentiation of the cells, because late passage cells recover their function recover after culturing them in 0.8 mM glucose [5].

Normally, glucose is catabolized via the oxidative phosphorylation pathway, which generates physiologic levels of reactive oxygen species (ROS). However, when chronically elevated glucose levels are present, this excess is shunted to other biochemical pathways, which in turn generate excessive ROS levels [4]. PDX-1 is a critical protein activator of the insulin gene promoter that plays an essential role in insulin synthesis. Chronically elevated ROS has been shown to reduce both *Pdx-1* and *insulin* gene expression and glucose-induced insulin secretion [9]. We have also shown that pharmacologic intervention of oxidative stress reduces markers of oxidative stress 4-hydroxynonenal (4-HNE and 80-HDG) and supports recovery of glucotoxic beta cell dysfunction [10]. Most recently, we showed that Kelch-like ECH-associated protein 1 (KEAP1):nuclear factor erythroid-derived 2-related factor (Nrf2) complex plays a central role in defending beta cells from the adverse effects of hyperglycemia-induced oxidative stress in high-fat diet–fed Zucker rats [13]. Nrf2 is an important intracellular protein that often is referred to as the master regulator of antioxidant gene expression [14-18]. Under quiescent conditions, Nrf2 is bound to KEAP1 in the cytoplasm, where it is cycled into proteasomes for degradation. In the face of oxidative stress, Nrf2 dissociates from KEAP1 and translocates to the nucleus, where it activates antioxidant genes (Fig. 1) to synthesize antioxidant enzymes for the purpose of neutralizing excessive levels of ROS. In the current study we examined gene expression levels of *insulin*, *PDX-1*, *Nrf2*, and *hemoxygenase-1* (*HO-1*) gene expression for 45 weeks during the development of glucose toxicity in HIT-T15 cells.

**Figure 1.**
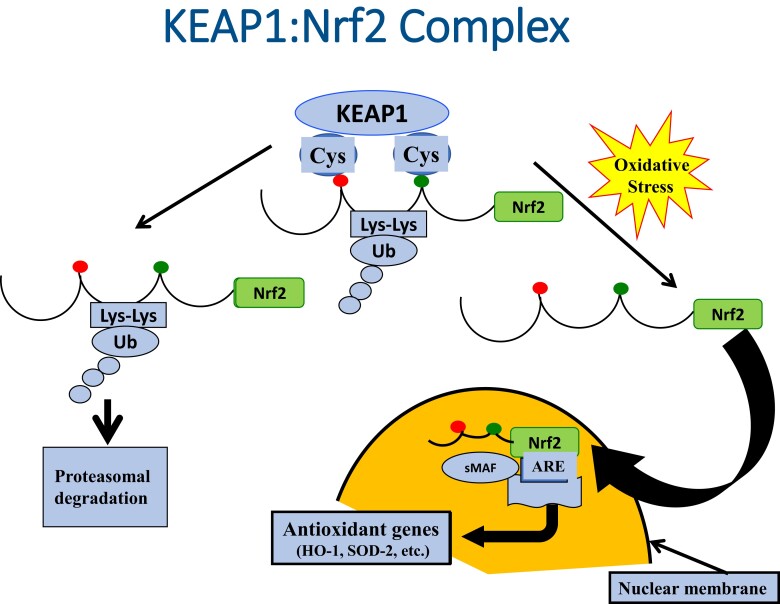
The KEAP1:Nrf2 complex. Nrf2 (nuclear factor erythroid-derived 2-related factor) is generally considered to be the master regulator of antioxidant gene transcription [14-18]. Under quiescent conditions, Nrf2 is bound by KEAP1 (Kelch-like ECH-associated protein 1) in the cytoplasm, which ushers Nrf2 to proteosomes for degradation. However, in the face of oxidative stress, Nrf2 dissociates from KEAP1 and enters the nucleus, where it serves as a key activator of the promoter for antioxidant genes.

## Materials and Methods

### HIT-T15 Cells

We began these studies with passage 70 (a passage defined as 1 week of subculture before splitting the cells into a further culture) and proceeded through passage 120, or 45 weeks of subculturing. P-70 cells were initially cultured in media containing either 0.8 mM or 16.0 mM glucose. These concentrations were selected based on previously published glucose dose–insulin response curves [1] to represent physiologic and supraphysiologic glucose conditions. The medium used was RPMI 1640; the medium was changed every midweek, and the cells were split each week to create the new passage number. The primary goal was to examine the levels of *Nrf2* gene expression using quantitative reverse transcription-polymerase chain reaction (qRT-PCR) measurements. We also examined mRNA levels of *insulin*, *Pdx-1* and *HO-1* (*hemoxygenase-1*).

### RNA Isolation and Quantitative RT-PCR Analyses

Forty-eight hours prior to RNA harvest, 5 million cells from each sample were subcultured in 11.1 mM glucose RPMI media. Total RNA was extracted using the RNeasy Mini Kit (Qiagen, Valencia, CA). Complementary DNA (cDNA) was then synthesized from 1 µg of total RNA using a QuantiTect Reverse Transcription Kit (Qiagen, Valencia, CA). PCR primers and probes for golden hamster *Insulin*, *Pdx1*, *Nrf2*, and *HO-1* were designed using Primer Express 3.0 software (Applied Biosystems). The sequences were as follows: *B-actin* forward, GGCTCCCAGCACCATGAA; B-actin reverse, GCCACCGATCCACACAGAGT; *B-actin* probe FAM-AAGATCATTGCTCCCCCTGAGCGC-TAMRA; Tbp forward, CTTCGTGCCCGAAATGCT; Tbp reverse, TCCGAGGCTCTCTTATTCTCATG; Tbp probe, FAM-ATCCCAAGCGGTTTGCTGCTGTCA-TAMRA; Ins forward, CCCTGCCCAGGCTTTTG; Ins reverse, CCAGGTAGAGTGCCTCCACAA; Ins probe, FAM-CAACCAGCACCTTTGTGGCTCCCA-TAMRA; Pdx1 forward, CCGCTGCCACCATGAAC; Pdx1 reverse, GGGCCCCTCTGGAATGC; Pdx1 probe, FAM-ACTATGCGGCCACACAGCTCTACAAGGA-TAMRA; Nrf forward, CCAGCACATCCGGACAGAT; Nrf reverse, TGCATGCAGTCTTCAAAGTACGA; Nrf probe, FAM-CCAGCTATTCCCAGGTGGCCCAC-TAMRA; HO1 forward, GACAACCCCACCAAGTTCAAG; HO1 reverse, GCTCCTCGAACAGCTCAATGT; HO1 probe, FAM-CTCTATCGCGCTCGCATGAACACTCTG-IBFQ. The relative abundance of the genes of interest was quantified using a TaqMan Gene Expression Master Mix (Applied Biosystems, Carlsbad, CA) with StepOnePlus Real-Time PCR System (Applied Biosystems, Carlsbad, CA). The cycling conditions comprised a 2-minute incubation hold at 50 °C for optimal uracil-DNA glycosylase activity and a 10-minute hold at 95 °C for polymerase activation, followed by 40 PCR cycles each comprising 15 seconds at 95 °C and 60 seconds at 60 °C for annealing and extension. Gene expression results were normalized to both B-actin and Tbp mRNA levels as per the method described in Vandesompele et al [19]. B-actin and Tbp were selected as the most stable from a GeNorm experiment involving 4 reference genes.

### Statistics

Statistical comparisons were made using 2 methods. In 1 method the mean of mRNA data obtained from 3 consecutive subcultures of passages before, after, and at a given time point (eg, mean data for P-80 were derived from individual data at P-79, P80, and P-81) to establish culture time epochs. These data were examined using 2-tailed comparisons with analysis of variance and the Tukey test, where *P* < .05 was considered to be statistically significant. In addition, Pearson's product moment correlational analysis was used to examine the biphasic slope (initially negative and later positive) observed when plotting passage number and Nrf2 mRNA levels.

## Results

**Figure 2.**
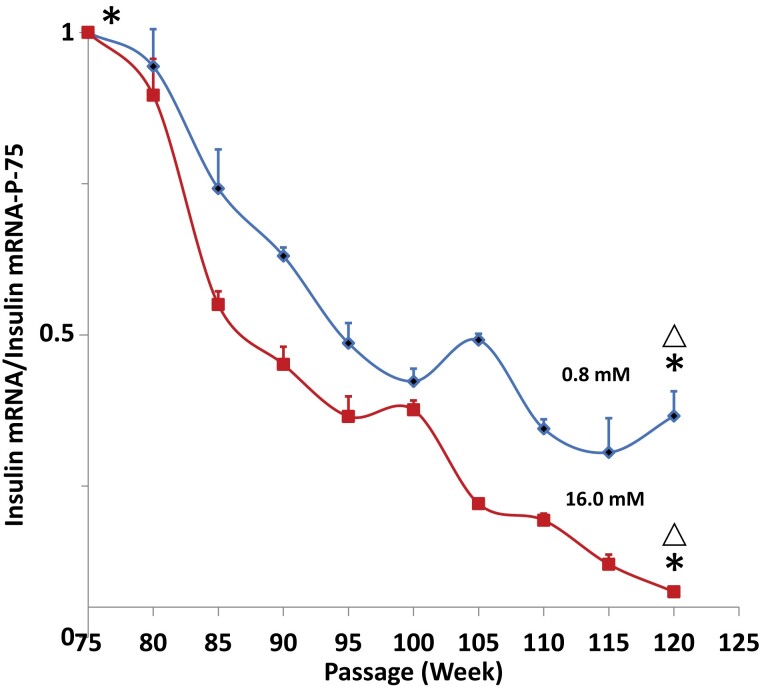
Insulin mRNA levels of HIT-T15 cells in RPMI 1640 media containing 0.8 mM glucose (blue line) or 16 mM glucose (red line). mRNA levels initially decreased but then stabilized at passage 105 in cells cultured under 0.8 mM glucose conditions. Asterisks indicate that mRNA levels at P-120 were significantly lower than those at P-75 for both 0.8 mM and 16.0 mM glucose concentrations. Triangles indicate that mRNA levels were significantly different at P-120 for the 0.8 mM and 16.0 mM concentrations (see “Results”).

**Figure 3.**
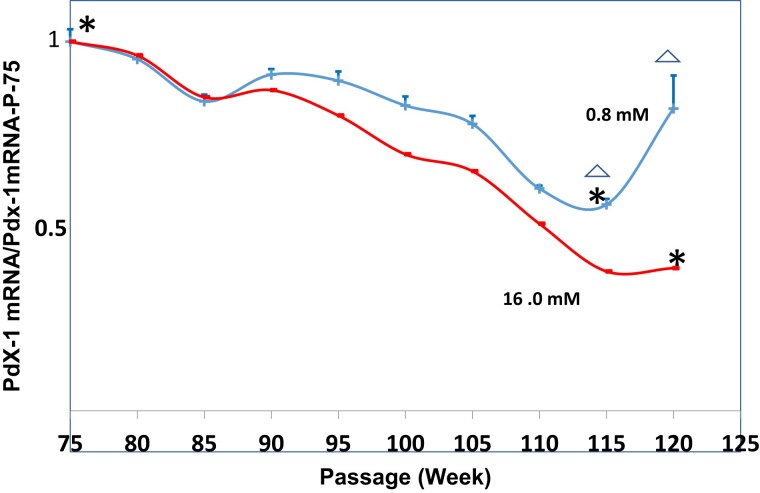
Pdx-1 mRNA levels of in HIT-T15 cells cultured in RPMI 1640 media containing 0.8 mM glucose (blue line) or 16 mM glucose (red line). mRNA levels initially decreased but then stabilized and increased by passage 120 in cells cultured under 0.8 mM glucose conditions. Asterisks indicates that mRNA levels were significantly lower for both the 0.8 mM glucose concentration (P-115) and the 16.0 mM glucose concentration (P-120) compared with P-75 levels. Triangles indicate that mRNA levels increased for the 0.8 mM glucose concentration at P-120 compared with those at P-115.

Insulin mRNA levels in HIT-T15 cells declined over time in both the low glucose (0.8 mM) and high glucose (16.0 mM) conditions. The mRNA level for the beginning passages was 1.00 ± 0.03 (Fig. 2). Cells cultured in both the 0.80 and the 16.0 mM concentrations underwent decreases in mRNA levels. However, the mRNA levels cultured in 16.0 mM glucose decreased to lower levels (P-120; 0.08 ± 0.01) than those in the cells cultured in 0.80 mM glucose (P-120; 0.37 ± 0.04; *P* < .001). The pattern of PDX-1 gene expression in HIT-T15 cells was similar to the pattern observed with insulin mRNA. The mRNA level for P-75 was 1.00 ± 0.03 (Fig. 3). Expression of PDX-1 in both the low glucose and high glucose conditions declined to similar levels by P-115, but thereafter levels for the low glucose condition increased while expression in the high glucose condition did not increase (levels at P-115 for 0.8 mM = 0.60 ± 0.08 and for P-120 = 0.45 ± 0.09; *P* < .001).

**Figure 4.**
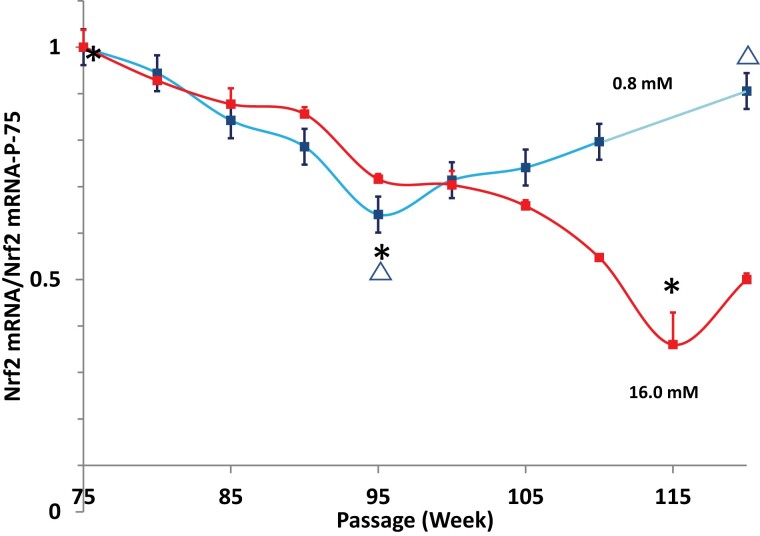
Nrf2 mRNA levels in HIT-T15 cells cultured in RPMI 1640 media containing 0.8 mM glucose (blue line) or 16 mM glucose (red line). mRNA levels initially decreased but then stabilized by passage 100 and then increased to nearly starting levels in cells cultured under 0.8 mM glucose conditions. Asterisks indicate that mRNA levels were decreased for both the 0.8 mM (P-95) and the 16.0 mM (p-120) glucose concentration conditions compared with those at P-75. Triangles indicate that mRNA levels for the 0.8 mM glucose condition were significantly greater at P-120 compared to those at P-95. Additionally, the statistically significant linear correlations were found from the downward slope data from P-75 through P-95 (r = −0.9859, *P* < .001) and from the upward slope data from P-100 through P-120 (r = +0.9930, *P* < .001).

**Figure 5.**
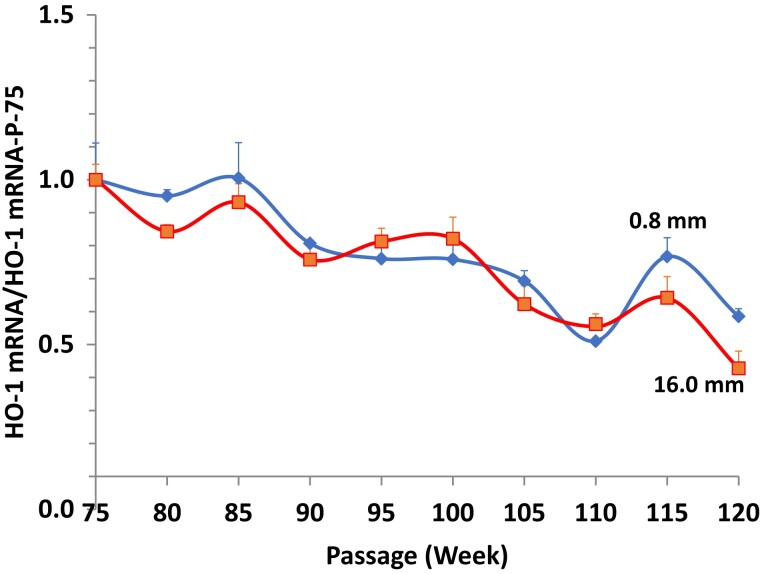
HO-1 mRNA levels in HIT-T15 cells cultured in RPMI 1640 media containing 0.8 mM glucose (blue line) or 16 mM glucose (red line). mRNA levels were generally similar in cells cultured with either 0.8 mM or 16.0 mM glucose but by P-115 tended to be slightly higher in cells cultured in 0.8 mM glucose.

Nrf2 gene expression in the cells cultured in media with 16.0 mM glucose or 0.80 mRNA declined over time (Fig. 4). However, even though Nrf2 mRNA levels in cells cultured under 0.8 mM glucose declined initially (P-95 vs P-75: 0.64 ± 0.02 vs 1.00 ± 0.03; *P* < .001), by passage 100 this trend had reversed such that Nrf2 mRNA under low glucose conditions increased to higher levels than in the high glucose condition (P-95 vs P-120: 0.64 ± 0.02 vs 0.91 ± 0.02; *P* < .001), which were nearly those of the starting cells cultured in 0.8 mM (P-120 vs P-75: 0.91 ± 0.02 vs 1.00 ± 0.03; *P* < .05). The correlation coefficient for the downward slope obtained when plotting Nrf2 mRNA level as a function of passage number P-75-95 was r = −0.9859, *P* < .001 and for the slope upward P-95-120 was r = +0.9930; *P* < .001. H0-1 gene expression in the low and high glucose conditions generally declined over time, although by passage 115 tended to be higher in cells cultured in 0.8 mM glucose (Fig. 5).

## Discussion

The relevance of oxidative stress to diabetes mellitus is that excessive levels of blood glucose can stimulate excessive levels of ROS in various metabolic pathways. Under physiologic conditions, glucose is primarily catabolized via glycolysis during which it generates physiologic amounts of ROS. However, in states of chronically high blood glucose levels, the glycolytic pathway becomes saturated with glucose, which is shunted to alternative pathways, including methylglycoxal/glycation, enediol formation, DAG/PKC activation, hexosamine metabolism, and sorbitol metabolism, all of which produce increasing levels of ROS and lead to a general state of oxidative stress [4]. The beta cell is especially susceptible to oxidative stress because, relative to other body tissues, it does not have appreciable amounts of 2 major antioxidants, namely, glutathione peroxidase and catalase. Consequently, the beta cell relies solely on superoxide dismutases and HO-1 to defend itself against intracellular peroxides and hydrogen peroxide. Notably, however, the product of superoxide dismutases includes hydrogen peroxide, itself an ROS, which consequently only increases the intracellular ROS load in beta cells. An overview of this beta cell–specific problem is provided in Robertson [4].

We have previously reported a study involving an animal model of diabetes, the male ZDF rat, which becomes promptly hyperglycemic when it is fed a high-fat diet (HFD; 13). ZDF beta cells initially met the challenge of hyperglycemia by secreting increasing amounts of insulin. However, this response was short-lived so that after 7 to 9 days of HFD the animals’ beta cells began to undergo structural damage, as shown by transmission microscopy, due to accumulation of intracellular ROS, as demonstrated by the fluorescent cellular marker of oxidative stress, 4-HNE. However, in rats fed a HFD for 9 days and then returned to regular diet for 2 weeks, the increase in 4-HNE and structural damage reversed and became undetectable. Importantly, this cellular repair was shown to be subsequent to appearance of Nrf2 in the nucleus and an accompanying generation of the antioxidant HO-1. There was no evidence of changes in rates of apoptosis or cell replication in these studies.

The current studies were primarily designed to examine the behavior of Nrf2 gene expression during chronic oxidative stress. While it is known that there is a rapid response of the pre-existing cytoplasmic KEAP1:Nrf2 complex to release free Nrf2 for translocation to the nucleus, the timing of a possible related response of Nrf2 gene expression to replenish cytoplasmic Nrf2 has not been previously examined. Since such studies are not possible using an in vivo model such as the ZDF rat, we pursued them by studying the beta-cell line HIT-T15 over a period of 45 weeks. The results are consistent with our previous studies demonstrating the loss of insulin content and Pdx-1 levels over time when HIT-T15 cells are continuously cultured in supraphysiologic glucose concentrations [9, 10]. In the current studies we observed initial decreases in Nrf2, Pdx-1, and insulin mRNA levels. However, over time, levels of Nrf2 had increased by passage 100, followed by increases in and stabilization of Pdx-1 and insulin mRNAs by passage 105. In contrast, the cells cultured in media containing 16 mM glucose demonstrated a continual fall in Nrf2, Pdx-1, and insulin mRNA levels. Levels of HO-1 mRNA generally declined over time in cells cultured in both 0.8 and 16 mM but were slightly higher in the former.

In conclusion, the main purpose of this work was to examine the behavior of Nrf2, Pdx-1, insulin, and HO-1 gene expression in a beta-cell line during prolonged exposure to physiologic and supraphysiologic glucose concentrations. Nrf2 in cells cultured exclusively in 0.8 mM glucose had a biphasic effect, in which they initially underwent a decrease in Nrf2 gene expression but by passage 100 exhibited a progressive rise that nearly reached that of cells at passage 75. This was associated with a subsequent rise in Pdx-1 and insulin mRNA levels. We speculate that this time-related decrease and then increase of Nrf2 mRNA was prompted by gradual depletion of cytoplasmic Nrf2 stores, which served as a regulatory signal for increased Nrf2 gene expression to restore cytoplasmic Nrf2 levels.

## Data Availability

The data reported in this manuscript are retained in Dr. Robertson's personal files and available to the scientific community for perusal without reservation.

## Abbreviations

HFD: high-fat diet
*HO-1*: *hemoxygenase-1*
KEAP1: Kelch-like ECH-associated protein 1
Nrf2: nuclear factor erythroid-derived-2 related factor
qRT-PCR: quantitative reverse transcription-polymerase chain reaction
ROS: reactive oxygen species
